# Gaucher Disease: Transcriptome Analyses Using Microarray or mRNA Sequencing in a *Gba1* Mutant Mouse Model Treated with Velaglucerase alfa or Imiglucerase

**DOI:** 10.1371/journal.pone.0074912

**Published:** 2013-10-04

**Authors:** Nupur Dasgupta, You-Hai Xu, Sunghee Oh, Ying Sun, Li Jia, Mehdi Keddache, Gregory A Grabowski

**Affiliations:** 1 The Division of Human Genetics, Cincinnati Children's Hospital Research Foundation, Cincinnati, Ohio, United States of America; 2 Department of Pediatrics, University of Cincinnati College of Medicine, Cincinnati, Ohio, United States of America; 3 CCR Bioinformatics Core, Advanced Biomedical Computing Center Frederick National Laboratory for Cancer Research, National Institutes of Health, Bethesda, Maryland, United States of America; Weizmann Institute of Science, Israel

## Abstract

Gaucher disease type 1, an inherited lysosomal storage disorder, is caused by mutations in *GBA1* leading to defective glucocerebrosidase (GCase) function and consequent excess accumulation of glucosylceramide/glucosylsphingosine in visceral organs. Enzyme replacement therapy (ERT) with the biosimilars, imiglucerase (imig) or velaglucerase alfa (vela) improves/reverses the visceral disease. Comparative transcriptomic effects (microarray and mRNA-Seq) of no ERT and ERT (imig or vela) were done with liver, lung, and spleen from mice having *Gba1* mutant alleles, termed D409V/null. Disease-related molecular effects, dynamic ranges, and sensitivities were compared between mRNA-Seq and microarrays and their respective analytic tools, i.e. Mixed Model ANOVA (microarray), and DESeq and edgeR (mRNA-Seq). While similar gene expression patterns were observed with both platforms, mRNA-Seq identified more differentially expressed genes (DEGs) (∼3-fold) than the microarrays. Among the three analytic tools, DESeq identified the maximum number of DEGs for all tissues and treatments. DESeq and edgeR comparisons revealed differences in DEGs identified. In 9V/null liver, spleen and lung, post-therapy transcriptomes approximated WT, were partially reverted, and had little change, respectively, and were concordant with the corresponding histological and biochemical findings. DEG overlaps were only 8–20% between mRNA-Seq and microarray, but the biological pathways were similar. Cell growth and proliferation, cell cycle, heme metabolism, and mitochondrial dysfunction were most altered with the Gaucher disease process. Imig and vela differentially affected specific disease pathways. Differential molecular responses were observed in direct transcriptome comparisons from imig- and vela-treated tissues. These results provide cross-validation for the mRNA-Seq and microarray platforms, and show differences between the molecular effects of two highly structurally similar ERT biopharmaceuticals.

## Introduction

Gaucher disease type 1, a common glycolipid storage disease, is caused by deleterious mutations in *GBA1,* which results in dysfunction of the lysosomal enzyme, glucocerebrosidase (GCase) and subsequent excess accumulation of glucosyl-ceramide (GluCer)/-sphingosine (GluSph) in various tissues [1]. In macrophages of the liver, spleen, and lungs, large accumulations of GluCer and lesser amount of GluSph lead to organ dysfunction. However, the molecular relationships of these pathological accumulations are poorly understood. In addition, the molecular pathogenesis of the variants of Gaucher disease with central nervous system and skeletal system involvement is elusive [2]. Gaucher disease type 1, the most prevalent variant in the Western World, has highly variable degrees of hepatosplenomegaly, cytopenias, and bone disease. The availability of mannose-terminated GCases that preferentially target macrophages, via the mannose receptor, has provided enzyme replacement therapy (ERT) for disease management, which has become the standard of care for the visceral disease of significantly affected patients [3], [4].

ERT reverses or ameliorates many of the manifestations of Gaucher disease type 1, including anemia, thrombocytopenia, hepatosplenomegaly and organ dysfunction, growth retardation and bone pain, and leads to dramatically improved quality of life for many patients [5], [6]. Pharmaco-kinetics and -dynamics of recombinant GCases have been evaluated in the 9V/null mouse [7]–[9]. This model is an analogue of human Gaucher disease that has been used to test various treatment modalities including ERT, substrate synthesis inhibition therapy, pharmacologic chaperone therapy [8], [10], and gene therapy [11]. Consistent with the human disease, ERT reduced GluCer storage in the visceral organs of these mice [7], [8]. However, little is known about the disease-related molecular events during the course of ERT compared to untreated individuals. End-stage gene expression profiles have been described in brains from neuronopathic Gaucher disease patients and mice [12], [13]. The global and macrophage activation gene expression profiles were defined in visceral organs of 9V/null mice [14], but have not been explored in a therapeutic setting.

Here, two structurally/biochemically similar FDA approved GCases, imiglucerase (imig, Genzyme/Sanofi) and velaglucerase alfa (vela, Shire/HGT) were compared for their molecular therapeutic effects in liver, lung, and spleen. Imig and vela have essentially identical *in vitro* kinetic properties, interactions with substrates and inhibitors [7], [15] and very similar crystal structures of the deglycosylated proteins [16], [17]. A significant difference between imig and vela is the number of mannoses contained in oligosaccharides on each of their respective 4 occupied N-linked glycosylaton sites. For vela expressed in human fibrosarcoma cells, the predominant number of mannoses is 9 [16], whereas for imig expressed in CHO cells, this is 3 [9]. These differences do not affect the *in vitro* stabilities or the kinetic properties of either enzyme [7], [9]. Pharmaco-kinetic and -dynamic studies showed essential similarities in tissue uptake and distribution between imig and vela using specific antibody assessments [7], [15]. Additionally, only minor differences in biochemical and histological effects with these drugs were found when 9V/null mice treated over a 12-fold range with imig or vela. In these imig- or vela-treated mice, the liver, lung, and spleen contents of GluCer and GluSph were not significantly different when compared at the same dose (5, 15, or 60 U/kg/wk) [7]. The molecular effects of these two drugs on gene expression in tissues have not been evaluated by transcriptomic analyses.

Here, a comprehensive study was conducted to evaluate the transcriptomic similarities and differences in the differentially expressed genes (DEGs) in the 9V/null model using both mRNA-Seq and microarray platforms. Also, the performance of the different analytic tools, i.e., ANOVA for microarrays, edgeR and DESeq for mRNA-Seq, were evaluated. The main objectives of these studies were to determine the effects on the Gaucher disease processes of imig and vela treatments, and to directly compare the molecular differences elicited by these two highly similar ERTs. The comparisons of the results with both platforms and analytic approaches also highlighted their advantages/disadvantages in identifying the DEGs profiles.

## Results

### Sample selection and data filtering

Comparative transcriptome analyses were performed in strain- and age-matched 9V/null mice that received weekly injections beginning at 20 wks of imig or vela (60 U/kg/wk for 8 wks, n = 31) or of saline (n = 17), and untreated WT mice (n = 12). The lungs, livers, and spleens were harvested one week after the last injection (28-wk old). Organ GluCer levels, as indication of correction of substrate accumulation in this model, were determined previously [7]. The ERTs resulted in almost complete correction of histology and GluCer accumulation in the liver, but the lung showed very little effect on these parameters. The ERTs led to partial normalization of splenic histopathology and GluCer levels [7].

The disease-related comparative transcriptomic changes as a result of ERT were evaluated by mRNA-Seq and microarray in the different tissues from 9V/null and WT mice. The data obtained from the two platforms were analyzed with the three appropriate analytic methods (see Methods and Fig. S1).

The biological replicates (n = 4–8) used here facilitated evaluation of the sample homogeneity in each tissue group. Principle Component Analyses (PCA) were used to show the overall structure of the data and how replicates grouped. PCA indicated general similarity in overall expression patterns within a tissue group. The PCA of microarray and mRNA-Seq showed distinct tissue separations in both platforms (Fig. S2). mRNA-Seq (55 samples) data showed 4 outlier samples of which 2 were from the spleen, and 1 each were from liver and lung. In addition to these, the microarray data identified 4 other splenic outliers. All the outliers were removed from the downstream DEG analyses. Of importance, including the outliers significantly affected identification of DEGs by mRNA-Seq and resulted in a small number of DEGs with random biological functions. This would have led to high false negative and positive discoveries (data not shown). Therefore, the PCA identification of outliers significantly reduced biased results, implying that identification and removal of outliers prior to down-stream DEGs detection is an important part of analyses of transcriptome data.

### Cross platform expression correlation

**Figure 1:**
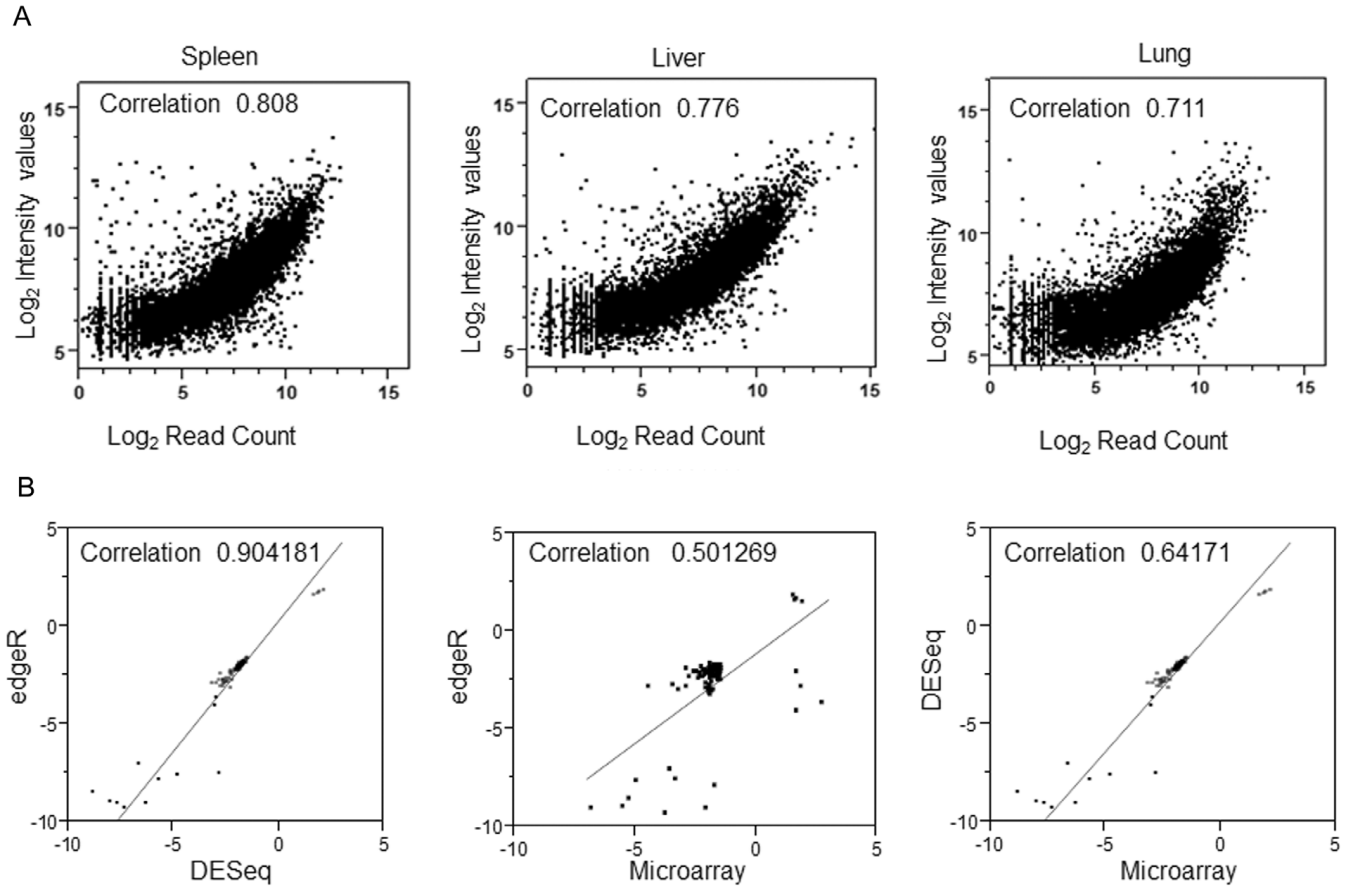
Correlations of microarray and mRNA-Seq and their DE analytic methods. (A) Correlation of signal intensity of saline treated 9V/null tissues in microarray platform with mRNA-Seq platforms. The panels show the (Log_2_) mRNA-Seq read counts for each gene plotted on the X-axis compared with the (Log_2_) intensities from the microarray data on the Y*-*axis. To avoid log of 0, 1 was added to each of the average counts prior to taking logs. The Pearson's coefficients (at the top of each panel) for each tissue show high correlation between the microarray and mRNA-Seq data. (B) Correlations of three DE analytic methods. edgeR and DESeq for mRNA-Seq and Mixed Model ANOVA for microarray were employed to pick a common subsets of genes from mRNA-Seq and microarray platforms. The genes that met the cut-off criteria (FDR  = 0.05, and a FC ≥ ±1.5) by all three DE methods were interrogated.

To validate the DEGs obtained from the microarray platform, mRNA-Seq was performed on the identical samples and analyzed by two different statistical methods. The analyses of the mRNA from the treated 9V/null mice are referenced to WT transcriptomes, which provided insight into the ERT effects on the disease-related molecular events. The mRNA-Seq and the microarray outputs are different; the former are discrete intensities of the read counts, while the latter are continuous intensity distributions. To perform correlations between the DEGs patterns from the two platforms, common sets of genes were selected, which were above the detectable threshold and common to both the platforms. Using these criteria, 17,157 genes were identified. The correlations (Fig. 1A) between the microarray and the mRNA-Seq data were assessed with the log-transformed values of the number of sequence reads mapped to each gene (mRNA-Seq) on the X-axis with the corresponding log-transformed intensity values (microarray) on the Y-axis. These two independent measures of transcript abundance were highly correlated with Pearson's correlation coefficients of 0.808, 0.776, and 0.711 (P<0.05) for spleen, liver, and lung, respectively, in saline treated 9V/null tissues as a representative example (Fig. 1A).

Fold-Change (FC) based comparisons were also performed to evaluate the ability of the two platforms to capture the different responses of gene expressions among three analytical methods under different condition. In 9V/null saline vs. WT data sets, the FC values of 105 DEGs were determined by all three analytical methods (Table S1), and were evaluated for correlation values (Fig. 1B). Spleen data sets were chosen as representative to compare these FC values. Several other subsets of genes were evaluated in each tissue under different treatment conditions (data not shown). In all cases the FCs of DEGs between the mRNA-Seq methods edgeR and DESeq were highly correlated (Pearson's correlation coefficient  = 0.904). Comparison of mRNA-Seq with microarray showed lesser correlations with Pearson's correlation coefficients  = 0.641 (DESeq and microarray) and 0.501 (edgeR and microarray) (Fig. 1B); i.e., the magnitude of FC values from the two platforms varied significantly.

### Comparisons of DEGs from microarray and mRNA-Seq

DEGs from saline-, imig-, and vela-treated 9V/null tissues were identified using three different analytical tools: Mixed Model ANOVA (microarray), and edgeR and DESeq (mRNA-Seq). The cut off criteria for selection of DEGs were based on a FC ±1.5 and an FDR (False discovery rate)  = 0.05. DEGs are listed in Tables S2, S3 and S4.

**Figure 2:**
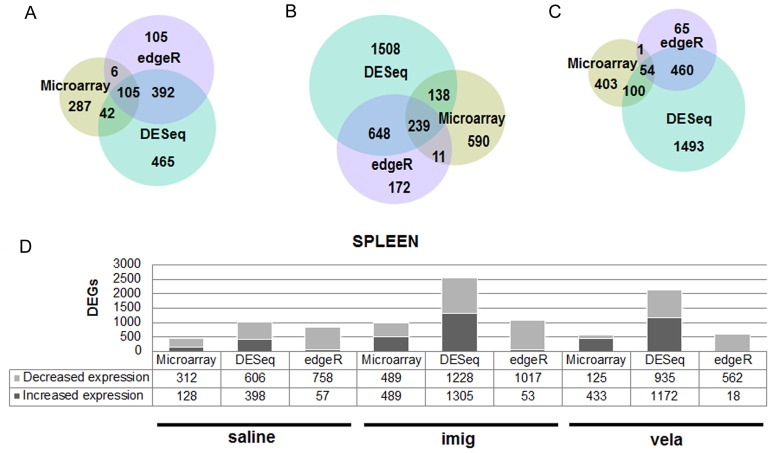
Comparisons of the DEGs between microarray and the mRNA-Seq. DEGs were identified in 9V/null vs. WT spleen by Mixed Model ANOVA (microarray) and DESeq and edgeR (mRNA-Seq). The colors indicate the analytic methods. (A) saline-treated, (B) imig-treated, and (C) vela-treated 9V/null spleen. (D) The number of DEGs in 9V/null spleen identified by the different analytic methods in the saline-, imig-, and vela- treated groups. The genes with increased expression levels are shown in dark grey and the genes with decreased expression levels are in light grey with the corresponding number of genes indicated below.

In the spleen, the DESeq and edgeR methods identified a larger number of DEGs as compared to microarray by Mixed Model ANOVA (termed microarray in the figures) (Fig. 2). These comparative analyses confirmed the greater sensitivity of DEGs analyses by DESeq and edgeR for mRNA-Seq compared with Mixed Model ANOVA for microarray [18].

In liver, significantly fewer DEGs were identified (Fig. S3). The DEGs were 36, 840, and 176 in 9V/null saline-treated vs. WT by Mixed Model ANOVA/microarray, DESeq and edgeR, respectively. Relative to saline-treated 9V/null livers, the numbers of DEGs were significantly changed post-imig or -vela treatment ranging from 68–95% decreased. In lung, DEGs were not significantly changed post-ERT (Fig. S4).

### Gene ontology and biological pathway analyses of spleen DEGs

Different analytical techniques were used to evaluate the transcriptome effects of imig or vela on the Gaucher disease processes using the saline-, imig-, or vela-treated mice compared to untreated WT. The functional categorizations were determined using DAVID. Based on the p value and the number of DEGs involved, cellular process genes contained 55–60% of the DEGs identified by the three analytic methods (Table S5). However, the numbers of DEGs in the different functional groups varied significantly between mRNA-Seq and microarray.

With the combination of the two mRNA-Seq analytic methods, most of the functional groups overlapped in the saline- and ERT-treated spleens with a few exceptions (Table S5). Despite of the few differences, the analyses of Gene Ontology (GO) terms were in general agreement between mRNA-Seq and microarray, leading to similar biological conclusions.

### Functional significance of the core splenic genes in saline-, imig-, and vela-treated samples

To evaluate the biological process, functions, and pathways in the ERT-treated mice, the DEGs identified by at least two of the three analytic methods were used. These were designated as core DEGs and are represented by the regions of intersections of the three-way proportional Venn diagram (Fig. 2A). The partial response of the spleen to ERT was the primary focus here. The number of core spleen DEGs were 545, 1923 and 615 with saline-, imig-, and vela- treatment, respectively.

**Figure 3:**
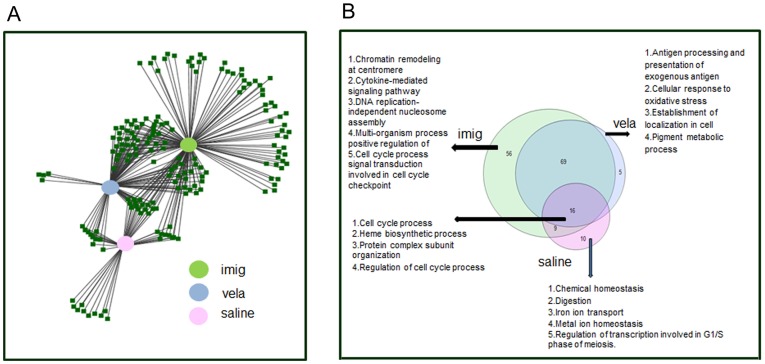
Functional classifications of the DEGs in spleen. (A) Functional relationship of spleen core DEGs associated with each treatment. An abstracted view shows the interaction of the biological functions by the core DEGs in 9V/null spleen compared with WT under different treatment conditions. The biological functions associated with the core DEGs from saline (pink node), vela (blue node) and imig (green node) treated 9V/null mouse spleens. Merged nodes indicate the shared functions between treatments. (B) 3-way Venn diagram presents the distribution of the biological functions by the core DEGs in spleen with different treatments. Each color represents a treatment as labeled. The GO were identified with DAVID. There were 16 functions common for 3 treatments. The unique functions for saline were 10, imig were 4, and vela were 56. The top biological functions are listed against each treatment.

The interactions between the significant biological functions under the different conditions are presented as an abstracted view developed with ToppCluster and Cytoscape (Fig. 3A). The numbers of common and unique functions associated with each big node/treatment condition are represented by the Venn diagram (Fig. 3B). There were 16 common functions shared by all three treatment conditions. These functions focused primarily on cell cycle processes and regulation, heme biosynthetic process, and protein complex organization. There were 56 unique functions associated with imig, 5 with vela, and 10 with saline treatment (Table S6). Imig and vela treatments shared 69 common functions. Most of the common functions were related to cell cycle regulation, mitochondrial ATP synthesis coupled with electron transport, regulation of programmed cell death, and regulation of protein ubiquitination. The genes in the cell cycle function which is ∼15% of the total DEGs in microarray and mRNASeq, were absent in vela-treated samples. The number of genes involved in cell death in imig-treated samples were twice those in the saline- or vela-treated samples. Some of the cell death genes (9 in saline, 8 in vela and 7 in imig) overlap with the autophagy genes, of which 7 are common between imig- and vela- treatment. They include *Bcl2l1*, *Bnip3l*, *Camp, Cox5a*, *Nqo1*, *Snca* and *Sod1*. In addition, imig-treated spleen has *Sphk1* and saline-treated spleen has both *Sphk1* and *Usp1*.

New functional groups, e.g., cellular growth and proliferation and immune cell trafficking, were identified in the post-treatment groups, suggesting relationships to disease repair mechanisms. In the saline-, vela- and imig-treated spleens, there were 62 (11.4%), 80 (13%), and 98 (9.5%) hematopoietic DEGs, respectively. Of these, 32 were common to imig-, vela-, and saline-treated spleen and 17 (53%) were in the myelopoietic gene cluster (Table S7). All the common hematopoietic genes had decreased expression levels in both enzyme- and saline- treated conditions, except for *Jak3*.

Apart from its hematopoietic function *Jak-3* is an important component of the JAK/STAT signaling pathway, and additional genes in this pathway showed abnormal expression. For example, the *Bcl2-like* gene that is involved in a wide variety of cellular activities had decreased expression levels in ERT and saline treated spleens. *Stat3* levels were increased by treatment with either enzyme, whereas *Socs* (suppressor of cytokine signaling) showed increased expression levels only in the imig-treated spleen.

The *GATA1* (globin transcription factor 1) and *PU.1/Sfpi1* (spleen focus forming virus pro viral integration oncogene) are the two DEGs that are lineage specification genes for the erythropoiesis and myelopoiesis lines, respectively. *Gata1* was within the core DEGs in spleen (Table S2) and showed decreased expression levels with either enzyme- or saline-treatment, but a greater decrease was found in the imig-treated spleens. In comparison, *Pu.1/Sfpi1* (Table S2) was identified by microarray only and showed increased expression levels in saline-, imig-, and vela- treated samples. Ten DEGs (Table S8) from saline, imig, and vela spleens interact with *Gata1* and *Pu.1*. All these interacting genes, except for *CD1d2,* showed decreased levels of expression. These indicate an imbalance between the lineage specifications with a repression of the erythropoietic line and an enhancement of the myelopoietic line.

**Figure 4:**
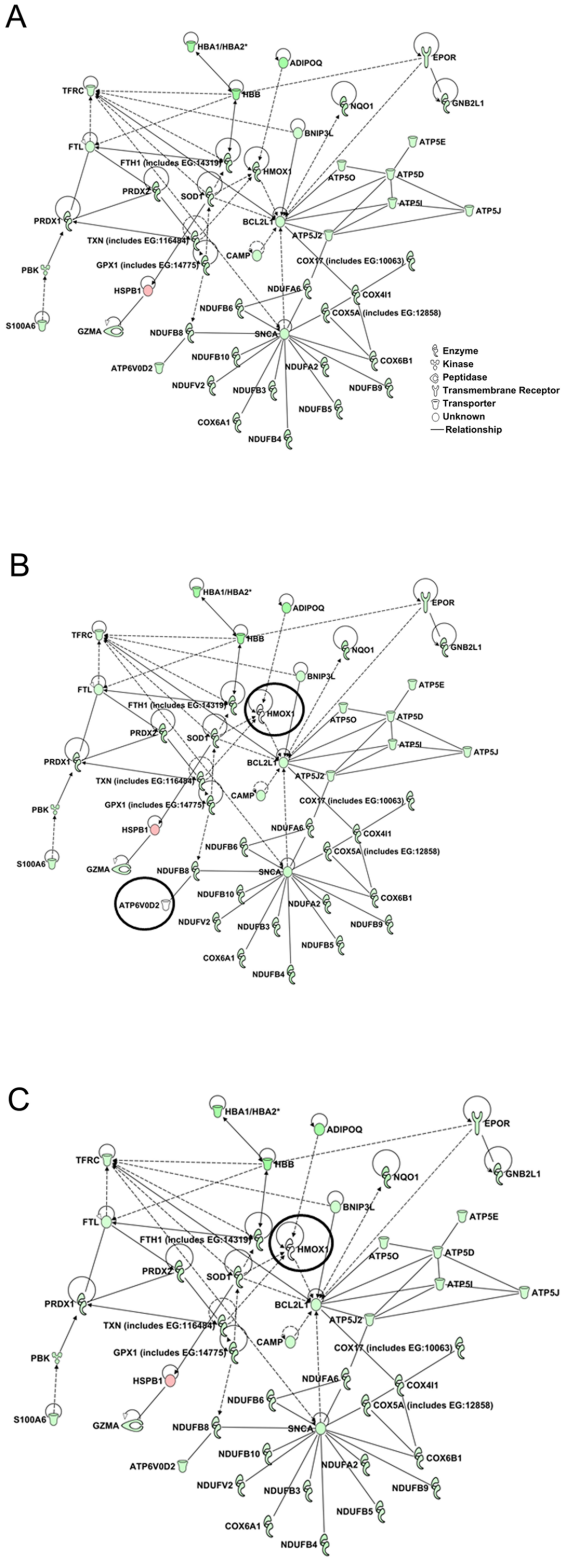
Spleen core DEGs forming network of mitochondrial dysfunction, oxidative phosphorylation, and ubiquinone biosynthesis. The network consists of 50 mitochondrial genes related to dysfunction, oxidative phosphorylation and ubiquinone biosynthesis. Genes colored with green or red indicate altered expression in saline-treated 9V/null spleen. Genes circled in black indicate the expression at WT level. (A) In the saline-treated 9V/null spleen, all genes in the network were abnormally expressed, shown in green or red. (B) The expression level of genes in imig-treated spleen. The ATPase and heme oxygenase circled in black were at WT levels. (C) In vela-treated spleen the expression of ATPase (circled in black) was at WT levels. Red indicates expression above normal and green indicates expression below normal levels.

The DEGs involved in the functional groups such as cell growth and proliferation, cell cycle, heme metabolism, and inflammation are altered during the course of ERT in Gaucher disease spleen. Significant number of DEGs identified were associated with mitochondrial dysfunction, oxidative phosphorylation and ubiquinone biosynthesis pathways. The DEGs in these pathways were combined to form a network of 45 DEGs in saline-treated spleens (Fig. 4A). All had decreased expression relative to WT, except for *Hspb1*. Treatment with imig (Fig. 4B) showed a return to WT levels in only two genes, *Atp6v0d2* (ATP synthase, H+ transporting, lysosomal 38 kDa, V0 subunit d2) and *Hmox1* [hemeoxygenase (decycling) 1]. Treatment with vela (Fig. 4C) had a similar effect only on *Hmox1*. FC values of those DEGs are in Table S9.

The protein ubiquitination pathway was unique to imig-treated spleen (Table S10). This pathway plays a major role in the degradation of short-lived or regulatory proteins involved in a variety of cellular processes, including cell cycle, cell proliferation, apoptosis, DNA repair, transcription regulation, cell surface receptors and ion channels regulation, and antigen processing. Regulation of *eIF4* (eukaryotic translation initiation factor 4) and *p70S6K* (p70 ribosomal S6 kinase) signaling, which is important for cell cycle progression, and G (1) and translation regulation [19] was among the most significant pathways in vela-treated spleen, but was absent in imig-treated spleen (Table S10). These different biological functions and pathways in each treatment indicate the differences in the effects of these two biopharmaceuticals at the molecular level derived either from elicited tissue reactions by imig or vela themselves or indicate differential time-dependent effects of the two enzyme treatments.

DEGs in 9V/null spleen identified several nuclear genes important to mitochondrial function and are associated with heme biosynthesis, including δ-*Alas2* (δ-aminolevulinic acid synthase 2), *Glrx5* [*glutaredoxin 5* homolog (S. cerevisiae)], *Slc25a38* (solute carrier family 25, member 38) and solute carrier *Slc25a39* (Table S11) [20]. These genes showed decreased expression levels relative to WT in ERT and saline-treated 9V/null spleens.

Amongst the transcription factors, *My*c (myelomatosis viral oncogene homolog) and *Mycn* (myelomatosis viral oncogene homolog neuroblastoma derived) were significantly altered in saline-, imig- and vela-treated spleens. The protein encoded by *Myc* plays a role in cell cycle progression, apoptosis and cellular transformation. It functions to regulate transcription of specific target genes. In addition *Rbpjl* (recombination signal binding protein for immunoglobulin kappa J region-like); *Nf2l2* (nuclear factor erythroid derived 2-like 2) and *Nrf1* (nuclear respiratory factor 1) were unique to vela-treated spleens, whereas *E2f4* (E2F transcription factor 4) was unique to imig-treated spleens (Table S12). *Stat3* was a common transcription factor with a significant p value overlap across all treatment conditions.

### Functional correlation of spleen, liver and lung

Ingenuity Pathway Analysis was used to evaluate the correlations of the biological functions, canonical pathways, networks, and transcription factors involved in the core DEGs in the enzyme and saline treated spleen, liver and lung tissues. The hematological system development and function was the only pathway shared by the core genes from these three tissues in the saline-treated 9V/null mice (Table 1). In liver imig reduced the number of DEGs by 66% while vela decrease all genes to WT levels (Table 1b). In the spleen and the lung the number of DEGs in this pathway increased post ERT. Based on the p value by Fisher exact test the top functional categories in the spleen (Table 1a), liver (Table 1b), and lung (Table 1c) included cell death, cell growth and proliferation, cell cycle, and heme metabolism pathways, suggesting these groups to be the most significant functions associated with the Gaucher disease and ERT processes.

**Table 1:** Top functional categories of the core DEGs in spleen (a), liver (b) and lung (c) under different treatment conditions in 9V/null vs. WT.

a. Spleen									
	saline			imig			vela		
Functional Categories ^1^	# of DEGs^2^	(%)	P Value	# of DEGs^2^	(%)	P Value	# of DEGs^2^	(%)	P Value
Cell Death	162	29.72	3.85E-09– 2.12E-02	298	28.76	6.73E-14– 3.91E-03	196	62.22	1.38E-11– 8.60E-03
Hematological System Development and Function	95	17.43	3.56E-09– 2.12E-02	177	17.08	1.89E-07– 3.74E-03	144	23.41	1.14E-09– 8.60E-03
Cell Cycle	82	15.05	5.71E-08– 2.12E-02	157	15.15	4.17E-11 – 3.74E-03			
Hematopoiesis	62	11.38	3.56E-09– 2.12E-02	98	9.46	5.22E-07 – 3.45E-03	80	13.01	2.32E-09 – 8.60E-03
Connective Tissue Development and Function	45	8.26	1.73E-05– 1.88E-02	84	8.11	6.03E-07 – 3.85E-03			
Cell Morphology	40	7.34	8.61E-09– 1.73E-02						
Organ Morphology	34	6.24	8.97E-05– 1.27E-02						
Free Radical Scavenging	28	5.14	3.95E-07– 1.71E-02				42	6.83	8.19E-08 – 6.28E-03
Cardiovascular System Development and Function							13	2.11	2.75E-05 – 8.60E-03
Cellular Development							142	23.09	2.96E-07 – 8.60E-03
Cellular Growth and Proliferation				291	28.09	2.77E-11 – 3.78E-03	181	29.43	6.49E-09 – 8.60E-03
Cellular Movement				169	16.31	2.63E-07 – 3.82E-03			
DNA Replication, Recombination, and Repair				130	12.55	7.54E-10 – 3.74E-03			
Immune Cell Trafficking				107	10.33	2.63E-07 – 3.74E-03	85	13.82	3.62E-07 – 8.60E-03
Small Molecule Biochemistry	63	11.56	6.98E-08 – 2.12E-02				84	13.66	8.19E-08 – 8.60E-03

b. Liver									
	saline			imig			vela		
Functional Categories ^1^	# of DEGs ^2^	(%)	P Value	# of DEGs^2^	(%)	P Value	# of DEGs^2^	(%)	P Value
Cell Death	50	31.85	1.08E-06– 9.49E-03				12	52.17	1.59E-06– 1.01E-02
Cellular Movement	32	20.38	1.27E-09– 8.99E-03						
Immune Cell Trafficking	30	19.11	1.27E-09– 8.99E-03	9	17.65	2.10E-05– 1.81E-02			
Inflammatory Response	42	26.75	1.27E-09– 8.99E-03	12	23.53	2.10E-05– 1.84E-02			
Hematological System Development and Function	44	28.03	1.27E-09– 9.54E-03	15	29.41	2.10E-05– 2.15E-02			
Molecular Transport	45	28.66	1.42E-09– 8.75E-03	19	37.25	1.38E-06– 2.14E-02			
Lipid Metabolism	36	22.93	1.42E-09– 9.52E-03	14	27.45	1.38E-06– 2.14E-02			
Small Molecule Biochemistry	46	29.3	1.42E-09– 9.52E-03	24	47.06	1.38E-06 – 2.14E-02			
Hematopoiesis	5	3.18	2.44E-05– 8.75E-03						
Tissue Morphology	35	22.29	6.08E-07– 8.75E-03	5	9.80	2.80E-05– 1.84E-02	10	43.48	2.33E-05– 9.26E-03
Tissue Development	29	18.47	6.10E-06– 9.54E-03	13	25.49	2.10E-05– 2.15E-02			
Organismal Development				13	25.49	6.62E-05– 2.15E-02			
Cell Cycle							6	26.09	1.20E-05– 1.01E-02
Cellular Compromise							7	30.43	5.93E-06– 8.66E-03
Cellular Development				14	27.45	2.80E-05– 2.15E-02	12	52.17	1.69E-05– 1.01E-02
Cellular Growth and Proliferation				14	27.45	2.80E-05– 1.84E-02	9	39.13	1.69E-05– 1.01E-02
Lymphoid Tissue Structure and Development							5	21.74	2.33E-05– 1.01E-02

c. Lung									
	saline			imig			vela		
Functional Categories^1^	# of DEGs^2^	(%)	P Value	# of DEGs^2^	(%)	P Value	# of DEGs ^2^	(%)	P Value
Hematological System Development and Function	88	34.51	6.15E-29– 5.25E-05	118	57.00	5.08E-36– 2.71E-05	102	59.30	1.31E-33 – 2.10E-05
Inflammatory Response	88	34.51	7.21E-25– 5.43E-05	111	53.62	1.34E-32– 2.71E-05	95	55.23	8.72E-29– 2.10E-05
Cell-To-Cell Signaling and Interaction	74	29.02	6.00E-26– 4.49E-05	84	40.58	4.32E-23– 2.68E-05	87	50.58	1.57E-28– 2.11E-05
Cellular Movement	72	28.24	9.03E-30– 4.49E-05	95	45.89	5.08E-36– 2.71E-05	90	52.33	9.89E-34– 2.10E-05
Immune Cell Trafficking	70	27.45	9.03E-30– 4.77E-05	86	41.55	5.08E-36– 2.71E-05	84	48.84	9.89E-34– 2.10E-05
Cellular Function and Maintenance	68	26.67	1.93E-21– 2.83E-05	90	43.48	9.56E-26– 2.02E-05	77	44.77	1.01E-17– 2.00E-05
Tissue Morphology	63	24.71	9.36E-25– 3.64E-05	75	36.23	1.57E-25– 2.25E-05	70	40.70	1.56E-23– 2.09E-05
Antigen Presentation	55	21.57	1.18E-17– 3.32E-05	78	37.68	2.24E-25– 2.71E-05	66	38.37	7.26E-24– 1.42E-05
Tissue Development	54	21.18	2.35E-17– 4.93E-05						
Protein Synthesis	41	16.08	3.32E-17– 4.07E-05	40		3.63E-20– 2.36E-05			
Humoral Immune Response	32	12.55	3.32E-17– 5.96E-06	45		3.63E-20– 2.36E-05	34	19.77	1.11E-15– 3.94E-06
Cellular Development							81	47.09	2.38E-17– 2.09E-05

1. Functional categories and DEGs were analyzed with Ingenuity Pathway Analysis (IPA). The table shows the number of genes, percentage of the genes and the associated P value in each functional category. Fisher's exact test was used to calculate a P value.

2. DEGs, Deferentially expressed genes.

In the liver, a significant reduction was found in the number of DEGs (50%–100%) post-ERT compared to spleen in which the number of DEGs related to these functions increased post-ERT with either drug (Table 1a and 1b). Inflammatory response was a top functional group in the liver and included 42 genes with altered expression. Post-treatment, the expression of 30 genes (71%) reverted to WT levels in imig-treated liver and all 42 genes (100%) changed to WT level in vela-treated liver samples. The DEGs involved in the top biological functions associated with the 9V/null treated and untreated lungs were hematological system development, immunological disease, immunological response, and cellular growth and maintenance. There was no reduction in the number of DEGs in the lung post treatment; rather there was a significant increase in the number of DEGs in these functional groups relative to WT (Table 1c). The liver and lung shared 3 functional groups – Inflammatory response, tissue morphology, and tissue development. For the first two functions, 70%–100% correction to WT level occurred in the liver post-ERT.

Saline-treated spleen and liver shared three functional groups: cell death, hematopoiesis and small molecule biochemistry. In the spleen and liver several disease-related DEGs were shared and derived from 3 pathways, including mitochondrial dysfunction, oxidative phosphorylation and ubiquinone biosynthesis. In the untreated liver, there were 10 DEGs with abnormal expression in the network. Unlike the spleen, 100% correction of those genes to WT levels was observed in the liver post imig- or vela-treatment (Table S9). The FC values of the DEGs in the liver and spleen show a differential molecular response to each ERT that is unique to each tissue and is concordant with the GluCer data in 9V/null spleen and liver post-ERT [7]. Thus, the molecular responses in the liver and spleen correlated with their biochemical changes.

### Molecular changes between imig and vela treatment

**Figure 5:**
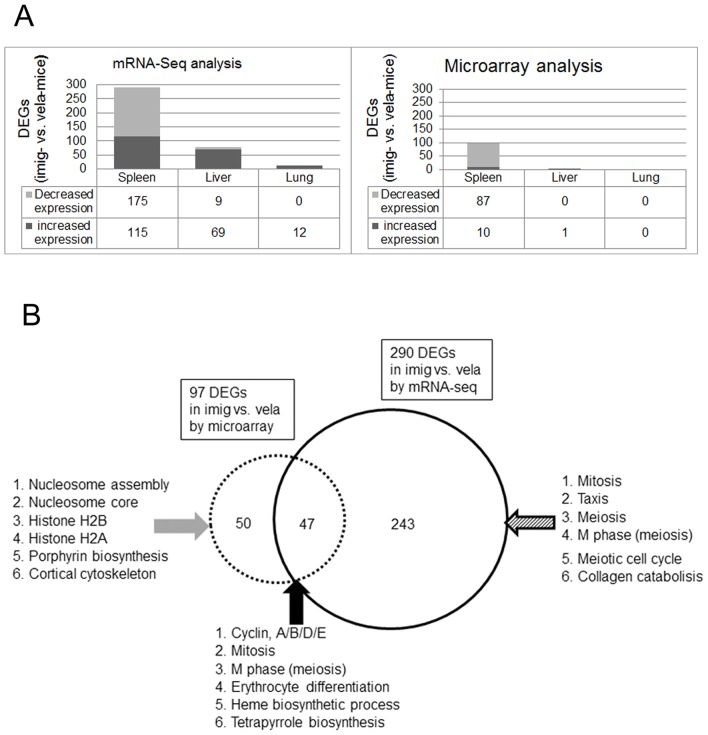
Comparative analyses of the DEGs identified by direct comparison of imig- vs. vela-treatment. For the analyses in this figure imig- and vela- treated samples were directly compared without normalization to WT gene expression in the corresponding tissue. (A) The Y-axis represents the number of DEGs in imig-treatment compared to the number with vela-treatment. The X-axis represents the different tissues. Three times more genes were detected by mRNA-Seq than that by microarray analysis. The number of genes are color coded for increased expression (dark grey) and decreased expression (light grey). Liver showed smaller DEG differences. In lung, The number of DEGs in imig- and vela-treated samples were not different. (B) Common and unique DEGs identified by microarray and mRNA-Seq in spleens. The Venn diagram of DEGs in the spleen compares the number of identified DEGs from microarray and mRNA-Seq that were different in imig- vs. vela-treatment. Compared to vela-treated spleen, 50 and 243 unique genes were identified in microarray (left) or mRNA-Seq (right) data sets in the imig-treated spleens. Forty seven genes (intersection) were common to both platforms. The GO annotation was performed using DAVID and the number of increased and decreased DEGs in the top functions identified by IPA are listed in Table S13.

The above analyses were conducted to assess the disease-related molecular changes as a result of ERT in the different tissues. To explore the potential molecular effects of two similar, but different, biopharmaceuticals, direct comparison were conducted with the transcriptomic profiles of imig- vs. vela-treatment. For these analyses, only the imig- and vela-data sets were used without reference to the WT data set. This direct comparison of imig- and vela-treatments would enable the detection of potential differences at molecular level between the effects of the two enzymes. mRNA-Seq analyses (DESeq statistics) showed 290, 78 and 12 more DEGs in imig compared to vela- treated spleens, livers, and lungs. Similarly, microarray analyses identified 97, 1, and 0 DEGs in the respective tissues (Fig. 5A). Compared to vela-treated tissues, imig-treated spleen, liver, and lung showed increased expression (by mRNA-Seq) by 40% (115 of 290) in spleen, 88.5% (69 of 78) in liver, and 100% (12 of 12) in lung of the DEGs (Fig. 5A).

### Commonality of imig vs. vela DEGs identified by microarray and mRNA-Seq

Direct comparison of the imig- and vela-treated spleen identified 47 DEGs, which were common by both microarray (47/97) and mRNA-Seq (47/290) analyses (Fig. 5B and Table S13). There were 50 or 243 unique DEGs detected with microarray or mRNA-Seq (Fig. 5B and Table S13). Among the 47 common DEGs, the most significant functional groups were related to the cell division/proliferation (32%) and the hematopoietic systems (11%) (Fig. 5B and Table S13). Interestingly, the unique DEGs in either microarray or mRNA-Seq also shared the same functional groups associated with the 50 common DEGs in both platforms. These groups included cell division/proliferation (24% in microarray and 49% in mRNA-Seq DEGs) and the hematopoietic system (26% in microarray and 7% in mRNA-Seq DEGs) (Fig. 5B and Table S13). The results suggest that the cell division/proliferation and hematopoietic systems as the predominant functions in the spleen that were altered in imig- vs. vela-treatment groups. mRNA-Seq analyses also identified inflammatory/macrophage response genes (10%) (Table S13). Taken together, three functional categories were most significant in the direct comparison of imig vs. vela treatment: 1) cell division/proliferation (imig/vela <1.5 FC, *p* = 2.03e-06 to 2.67e-02), 2) hematopoietic system (imig/vela <1.5 FC, *p = *2.92e-10 to 2.53e-02), and 3) inflammatory/macrophage (imig/vela >1.5 FC, *p = *5.53e-12 to 5.94e-03).

**Figure 6:**
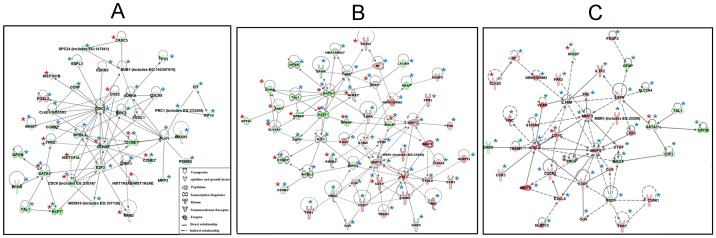
DEGs in splenic networks. The networks were generated using IPA software and were from the direct comparisons of imig- vs. vela-treated data sets without normalization to WT. The pathway included DEGs with decreased expression (imig/vela, green symbols) and the DEGs with expression level-increased (imig/vela, red symbols). The gene symbols and their interactions are as indicated. (A) The cell division/proliferation network is composed from total 42 DEGs determined by microarray (12 genes, red star) and mRNA-Seq (37 genes, blue star) which includs 7 common genes (blue and red stars) (see gene list in Table S14a). A general decrease in DEG expression levels was found in cell division/proliferation network from imig-treated vs. vela-treated spleen. (B) Hematopoietic system network was composed of total 54 DEGs determined by microarray (16 genes, red star) and mRNA-Seq (49 genes, blue star). Among them, 11 were common genes (red and blue stars) (Table S14b). (C) Inflammatory response/macrophage network was composed of total 41 DEGs determined by microarray (5 genes, red star) and mRNA-Seq (40 genes blue star), of those 4 were common genes (red and blue stars) (Table S14c).

The DEGs involved in these three functional categories were evaluated for network connections. The cell division/proliferation network contained 14 DEGs from microarrays and 36 from mRNA-Seq, 8 were detected with both platforms (Fig. 6A and Table S14a). The hematopoietic system network contained 16 DEGs from microarray and 49 from mRNA-Seq, of which 11 DEGs were detected by both platforms (Fig. 6B and Table S14b). This network had ∼50% of the DEGs either increased or decreased in the direct comparison of imig vs. vela (Table S14b). The inflammatory/macrophage response network contained total 41 DEGs. Among them 5 DEGs were detected by microarray, 40 by mRNA-Seq, and 4 detected by both platforms (Fig. 6C and Table S14c). Of 41 total DEGs detected by either or both platforms, 79% (32/41) were increased in imig- vs. vela-treated spleens. An additional 11 macrophage response-related genes were found in the mRNA-Seq data sets with increased levels in imig vs. vela, including *Arg2, Cd77, Cd44, Cd00lb, Cdl, Ifi204, Il1f9, Irg1, Ifi204, Mmp19, and Tarm1* (Table S14c).

The expression levels of *Gata1* and numerous other transcription factors involved in erythropoiesis were decreased in 9V/null mouse spleens treated with saline, imig, or vela (Table S7). These decreases were also evidenced in the direct comparison (without WT reference) of imig- vs. vela-spleens (Table S14), indicating a disrupted balance in erythropoiesis and myelopoiesis in the Gaucher disease process and its treatment. In addition, these DEG levels were more severely depressed in the imig-treated spleens compared directly to vela-treated spleens. These results demonstrate different molecular responses between two biosimilar GCases, imig and vela, during the ERT process in this Gaucher disease mouse model.

## Discussion

With improvement in technologies and analysis algorithms, microarray and mRNA-Seq hold great promise to reveal deeper insights into the fundamentals of gene expression variations in disease states and between therapeutics. mRNA-Seq platforms have several advantages compared with microarray, chief among which is its greater dynamic/detection range, particularly at low expression levels. Here, two different analytical methods (DESeq and edgeR) were applied in the analyses of mRNA-Seq data and compared with that from cDNA microarrays. These studies were designed to understand the molecular effects of Gaucher disease and of two biosimilar ERTs on the disease processes in different tissues and to compare the different platforms and statistical approaches to their analyses. An unexpected result was the transcriptomic effect differences between the two biosimilars, imig and vela since they differ by only a few mannosyl residues on their N-linked oligosaccharides. By direct comparison of these two biosimilars without any normalization to the WT or saline-treated 9V/null mice, differences were clearly evident in the transcriptomes. The molecular differences imply differential mechanisms and molecular pathways in the therapeutic responses of Gaucher disease to these two biopharmaceuticals.

### Comparison of two gene expression platforms

mRNA-Seq allows a comprehensive evaluation and quantification of all subtypes of RNAs in cells or tissues [21]. mRNA-Seq technology can detect transcripts expressed at low levels [22] and permits the identification of unannotated transcripts and new spliced isoforms [21], [23]. Previous transcriptomic studies using microarray relied on hybridization-based technologies, which were probe-based with limitations in detection range due to background noise and signal saturation [21]. This approach also was limited to the catalogue of molecules represented by the probes and prespecified targets [24]. The cross-hybridization and detection levels that effect the accuracy of microarray gene expression estimations are not relevant to mRNA-Seq [25]. Several studies have compared mRNA-Seq and microarray. These include the proof of principal of the NGS platforms [21], [26] and analyses methodology development [27].

Several comparison studies of mRNA-Seq and microarrays have addressed different biological questions, i.e., the relative merits of the two techniques and their inherent biases [22], expression differences between tissue types that focused on the technical variance in NGS technology [18], genomics study comparing the effect of Aristilochic acid on rat kidneys [28], and transcriptional profiling of cerebro-osteodysplasia [29]. Such studies showed mRNA-Seq was more sensitive than microarray, but similar gene expression patterns were obtained with both platforms. Results here with identical RNA samples showed only ∼50% overlap in DEGs, but the biological interpretation was largely consistent between the two platforms. Standard tools have been established for the analyses of transcriptomes from microarray data. Importantly, comparisons of mRNA-Seq and microarray data are critical because of the existence of a plethora of data from microarrays that could continue to be used for future studies as mRNA-Seq becomes standard. Here, a strong congruence was found between the different platforms (Fig. 1A). The relationship was not quite linear, as there was a slight compression in the microarray data at high expression levels, but the vast majority of the derived expression values are similar. The scatter increases at low expression, which is not surprising, as background correction methods for microarrays are complicated when signal levels approach noise levels. The present results also demonstrated that mRNA-Seq platform was more sensitive than the microarray platform and identified approximately three times more DEGs than microarray using identical samples. mRNA-Seq has a larger dynamic range of expression levels over which transcripts can be detected, particularly the genes with low expression level. In contrast, DNA microarrays lack sensitivity for genes expressed either at low or very high levels and therefore have a much smaller dynamic range (one-hundredfold to a few-hundredfold). This increased dynamic range of mRNA-Seq facilitated the comparisons of genes involved in the disease processes in DEGs with low levels of expression and similar DEGs in the comparative studies of the biosimilars.

### mRNA-Seq data analysis tools – edgeR and DESeq

The output from NGS mRNA-Seq gives a discrete intensity of read counts and Poisson's distribution is most suitable for mRNA-Seq data [18], [29], [30]. To address the over dispersion problem, the model for the count data was addressed with a negative binomial (NB) distribution. Both edgeR and DESeq methods used here are based on the NB. As a parametric distribution approach, the generalized linear model in edgeR package with NB was included to take into consideration the variability in sample replicates. In the model, treatment specific differences within a particular tissue were investigated as the main factor by controlling over-dispersed variability of biological samples as nuisance factor.

DESeq is similarly modeled on the NB distribution. The main difference between edgeR and DESeq is the different approaches to estimate dispersion parameter [31], [32] and normalization procedures. The edgeR method uses quantile adjustment, while DESeq adjusts counts by scaling [31]–[33]. Our choice of these DE methods was based on the literature and robustness of these two methods compared with other tools.

There was reasonable concordance of DEGs between DESeq and edgeR for the liver, lung, and spleen mRNAs, thereby facilitating identification of the tissue and treatment specific transcriptomic alterations by either method. Pearson's correlation coefficients of the FC were used to assess similarity between microarray/Mixed Model ANOVA, mRNA-Seq/DESeq and mRNA-Seq/edgeR. All three methods showed good agreement when using a subset of DEGs between all three tissues.

### Molecular responses to imig- vs. vela-treatment

Based on the previous biochemical and histopathological studies, the liver, spleen, and lungs of the 9V/null mice had complete, partial, or little response, respectively, to either imig or vela, and there were only small differences in these therapeutic effects of these biosimilars [7]. Here, the tissue effects to two nearly identical biopharmaceuticals were compared to the untreated and WT for their transcriptomic effects using mRNA-Seq and microarray platforms, and, importantly, the three standard analytic statistical approaches were compared. Surprisingly, substantial differences were found between the disease-related transcriptomic effects of the two drugs in addition to the differences between the technologies and analytic methods.

Biologically, both platforms indicate a tissue specific correlation of the DEGs as observed in the PCA plots where the liver, lung, and spleen are clustered into three distinct groups. Analyses of the biological functions, pathways and networks with the core spleen DEGs showed cell growth and proliferation, cell division, cell death, and heme metabolism are common functions across all tissues and treatment conditions in 9V/null spleen. The Gene Ontologies for the common DEGs suggest that the top functions, based on the number of DEGs involved and the p value, include cell growth and proliferation, cell death and survival, and inflammation. The unique genes in the imig and vela group mainly coincided with the same functional groups with some differences. The number of genes associated with the common functions is greater in the imig treated group compared to vela. This suggests that even though the two drugs are very similar structurally and functionally there are differences at the molecular level. Overall functional analyses suggest overlap of some significant canonical pathways, i.e., the oxidative phosphorylation, mitochondrial dysfunction and ubiquinone biosynthesis in the saline-treated 9V/null liver and spleen samples (Table S8). Interestingly, some of the mitochondrial dysfunction genes overlapped with the heme biosynthesis pathway. The heme biosynthesis pathway is a tightly orchestrated process that occurs in all cells [34]. In most eukaryotes, the first step in heme synthesis is the mitochondrial gene, δ-aminolevulinic acid synthase (δ-Alas), which catalyzes the reaction between succinyl-CoA and glycine to form δ-aminolevulinic acid, δAla. Defects in *δ–Alas2, Abcb7* [ATP-binding cassette, sub-family B (MDR/TAP), member 7], *Glrx5* [glutaredoxin 5 homolog (S. cerevisiae)] and *Slc25a38* (solute carrier family 25, member 38) are causal to different forms of sideroblastic anemias [35]–[37]. These exhibit mitochondrial iron overload and impaired heme synthesis. The solute carrier *Slc25a39* is important for maintaining mitochondrial iron homeostasis and regulating heme levels [37].

Mitochondrial dysfunction has been reported in lysosomal diseases in part due to the involvement of the autophagy/mitophagy system(s) [38], [39]. Recent studies suggest that mitochondrial dysfunction and subsequent ATP deficiency may be responsible for the neuronal impairment in Niemann-Pick Type C and Gaucher diseases [40], [41]. Mitochondrial dysfunction increases with aging and has been found in Parkinson's and other neurodegenerative diseases [42]–[44]. Indeed, heterozygotes for *GBA1* mutations occur with greater frequency in patients afflicted with Parkinson's disease [45] and there is a pathogenic relationship between GCase alterations, mitochondrial dysfunction, and Parkinson's disease [41], [46]–[48]. These observations and the current data support the involvement of altered mitochondrial function, hematopoiesis and myelopoiesis as important molecular processes in the progression of Gaucher disease.

Jak3, in the JAK/STAT pathway, is the only hematopoietic gene with increased expression in treatment with either enzyme or saline. Both STAT3, and SOCS have been recognized for their anti-inflammatory actions [49], [50]. The imig and vela ERT showed increased expression of both STAT3 and SOCS suggesting that a reduction of the lipid mediated increases of inflammatory immune response via this pathway. This provide a pathway for development of therapeutics for Gaucher disease, since involvement of JAK-STAT pathway and increases of the cytokines are evident.

### Function evaluation of DEGs in imig- vs. vela-treatment by direct comparison

Direct comparison of the transcriptomes from imig- vs. vela-treated spleen without reference to the WT data set identified total 90 genes involved in hematopoiesis. The majority (81/90) of these network genes were also detected in the imig-spleen normalized to WT controls (Table S14b), which indicates that the detected DEGs by imig- vs. vela- direct comparison represent the valid signals over the noise and their functional relevance. Surprisingly, 60% (54/90) of these network genes overlapped with the untreated spleen suggesting their disease-related origins. The disease-related genes from imig- vs. vela-spleens could be due to a “therapeutic-lag” from slowly disappearing/healing processes underlying imig treatment. Based upon the overlap with the untreated spleen (Table S14), the 90 network DEGs from imig- vs. vela-spleens can be assigned to disease-related (60%, 54 DEGs) and ERT-related (40%, 36 DEGs). Most of these ERT-related genes were cytokine and macrophage response genes, and their expression levels were altered, which indicates a relatively active status of inflammatory/cytokine genes in imig-treated spleen compared with vela-spleen.

The large numbers of inflammatory/macrophage response-related genes in the imig- vs. vela- treated spleen indicate different molecular events in the therapeutic pathways of these two highly similar biologics. Interestingly, 5 DEGs (imig vs. vela) that showed decreased expression were present in all 3 networks. They include *Gata1*, *Gfi1b*, *Tal1*, *E2f2*, and *Birc5* that encode regulatory proteins in erythroid lineage specification and cell division control. The direct comparisons in spleens identified increased cytokine/macrophage and decreased hematopoietic proliferation gene expression in imig-treatment relative to vela-treatment. Thus, two major molecular pathways were differentially responding in the spleen with either imig- or vela- treatment.

### The PU.1/GATA1 reciprocal effects

*Gata*1 expression levels were decreased across all 3 networks derived from imig- vs. vela-treated spleen (Fig. 6). GATA1 (GATA binding protein 1, globin transcription factor 1) is a zinc finger protein that is involved in lineage commitment for erythropoiesis [51], [52], megakaryocytopoiesis [53], and myelopoiesis [54], as well as in a variety of cell-cell signaling [55], cell development [56], and eosinophil differentiation [57]. The transactivation activities of GATA1 require interaction with Friend Of GATA (FOG)-1 cofactor [58] and other cofactors including EKLF, SP1, CBP/p300, LMO2, LDB1, RUNX1, FLI1, and PU.1 [59]–[61]. These cofactors constitute a complex network regulating erythropoiesis and megakaryocytopoiesis by promoting or repressing GATA1 activity [52], [62]. PU.1 is known to interact directly with GATA1 to regulate lineage specification during erythropoiesis and myelopoiesis. *Pu.1* was identified in this study only by the microarray platform in the spleen and showed increased expression levels under non-treatment (saline) and treatment (imig or vela) conditions. PU.1 is a key transcription factor that, with GATA 1, regulates the balance between the myeloid/erythroid pathways.

The mRNAs encoding other important regulatory proteins in erythroid development and proliferation control also were identified: transcription factor Tal1 [52], erythrocyte structural protein spectrin alpha (Spna1) [63], erythrocyte protein band 4.9 (Dematin/Epb4.9) [64], growth factor independent 1B (Gfi1b) [65], hemoglobin-alpha (Hba-a1/b1) [66], erythroid differentiation E2F transcription factors [67] and erythropoietin receptor (EpoR) [68]. EpoR is activated by GATA1 and functions as the receptor of EPO involved in EPOR/PI3K/AKT signaling pathway for cell erythroid proliferation [68], [69]. In addition, GATA1 regulated Eklf/Klf1 (for erythroid Kruppel-like factor 1) was down-regulated. Klf1 is a co-factor with GATA1 and SCL/TAL-1 for both primitive and definitive erythropoiesis [59], [70]. The decreased erythropoietic gene expression and increased myelopoietic gene expression indicates a reciprocal interaction of Pu.1/Gata1 expression and regulation in the spleen of Gaucher disease.

Indeed, increased expression levels of multiple inflammatory/macrophage activation genes correlated with increased expression of *Pu.1* in Gaucher disease. Here, 27 cytokine/macrophage genes in hematopoietic and inflammatory/macrophage networks had increased expression levels, which indicate the cellular and molecular events favored myelopoiesis in the spleen of 9V/null mice. Similar findings in our previous study showed many pro- and anti-inflammatory cytokines/mediators were up-regulated with macrophage proliferation in the visceral organs of 9V/null mice [14]. In addition, affected Gaucher disease patients had increased serum levels of pro-inflammatory cytokines, i.e., TNF-α, IL-6, IL-8, IL-1β, sIL-2R and anti-inflammatory cytokines i.e., IL-1rn, sCD14 [71]–[74], which could be the result of PU.1/GATA1 reciprocal effects in the erythropoietic/myelopoietic system and lead to a general lower- expression profiles of genes involved in erythroid proliferation and development [75]. These results imply important cellular/molecular mechanisms in the disease pathophysiologic process that may control the marrow suppression, particularly the megakaryocytes and erythroid precursors. The fundamental cellular/molecular mechanisms of this reciprocal expression of Pu.1/Gata1 and their roles in the pathophysiology of Gaucher disease are the subject of further study.

In conclusion, this study shows that NGS technologies are able to assess the transcript abundance at the whole genome level and their response to drug interventions. With continued cost reduction and improved analytical methods, NGS has begun to have a direct impact on biomedical discovery and clinical outcome [76], [77]. This provides great potential to advance our understanding in the biological mechanism. Commonly, comparisons are made in biological systems subjected to different stimuli or of normal and diseased states to elucidate the differences in the expression of genes that lead to altered endpoint phenotypes. Various statistical algorithms have been developed for identifying DEGs between different groups, and the choice of proper and robust method can have a profound influence on the interpretation of the transcriptome data. However, careful analysis and interpretation of the data should be taken. Using multiple methods and platforms in this study provides a validation for robust and convincing data output.

ERT in a Gaucher disease mouse model clearly demonstrated that both imig and vela achieved similar therapeutic effects at biochemical and histological levels [7], but at the molecular level their paths to normalization are different and tissue specific. Thus, these two structurally and functionally similar biopharmaceuticals had unexpected molecular effects leading disease correction.

## Materials and Methods

### Materials

The following were from commercial sources: imiglucerase (imig, Cerezyme®, Cambridge, MA) is a recombinant human GCase from overexpressing CHO cells; velaglucerase alfa (vela, VPRIV™, HGT/Shire, Cambridge, MA) is a gene-activated GCase from human fibrosarcoma cells. RNA Later and TOTALLY RNA kit (Ambion, Austin, TX). Trizol (Invitrogen, Carlsbad, CA). Affymetrix Mouse Gene 1.0 ST chip (Affymetrix, Santa Clara, CA). Illumina TruSeq RNA library preparation kit and Illumina HiSeq2000 (Illumina, Inc., San Diego, CA). Avadis® NGS software, Version 1.3.0, (Strand Scientific Intelligence, Inc., San Francisco, CA). JMP Genomics 5 (SAS Institute Inc., Cary, NC) and Ingenuity Pathway Analysis (IPA) (Ingenuity Systems, Mountain View, CA).

### Mice with point-mutated GCase

Knock-in mice with a *Gba1* point mutation encoding Valine (V) 409 for the WT Aspartate (D) on one missense allele and a null heteroallele [D409V/null (9V/null)] and WT littermates were of mixed, but matched, genetic backgrounds of C57BL/129Sv/FVB [7]. The CCHMC Institutional Animal Care and Use Committee (CCHMC IACUC) reviewed and approved these studies under protocol 7C02017. All mice were housed in the pathogen-free barrier facility and according to IACUC standard procedures at Cincinnati Children's Hospital Research Foundation. Mice were monitored daily and weighed weekly.

### Enzyme replacement/reconstitution therapy (ERT)

9V/null mice were injected weekly via tail-vein bolus with 60 U/kg of imig or vela for 8 wk [7]. Control 9V/null mice were injected with the same volume of saline (vehicle). Mice were sacrificed one week after the last injection, and lungs, livers, and spleens were collected for lipid (GluCer, GluSph) and RNA analyses.

### RNA sample preparation for microarray and mRNA-Seq

For total RNA isolation, the lungs, livers, and spleens were collected from untreated WT, and saline and enzyme treated 9V/null mice. The RNA sample sets were prepared by pooling of mRNA from three individual mice for each tissue type in a treatment cohort. Total RNA was isolated from the organs of control and imig and vela injected 9V/null mice (28 wk of age) and WT mice. Collected tissues were immediately immersed in RNA Later and RNAs were extracted using the TOTALLY RNA kit (lung and liver) or Trizol (spleen). Whole transcriptome analyses of identical aliquots of RNA were performed using the Affymetrix Mouse Gene 1.0 ST Array (microarray) and Illumina TruSeq RNA Sequencing kit (mRNA-Seq). Each treatment cohort was composed of 3 or 4 sets RNA samples: 2 to 3 single tissue RNA samples from individual mice and one pooled RNA of those individual samples.

Sample sets were as follows: 32 lung and liver sample sets included 4 each of imig, vela, or saline from 9V null mice and 4 WT controls for each tissue. Twenty-three spleen sets included 4 imig, 4 vela, 7 saline from 9V/null mice and 8 WT mice. Whole transcriptome analyses were performed using aliquots from identical sample sets applied to the Affymetrix Mouse Gene 1.0 ST chip (microarray) at the Gene Expression Microarray Core and the Illumina Hi-Seq2000 (mRNA-Seq) at the Genetic Variation and Gene Discovery Core of CCHMC [78]. Microarray hybridization and sequencing results from saline, imig, and vela treated spleen, liver, and lung from 9V/null mice were compared with untreated WT samples.

The microarray and mRNA-seq data set obtained from the 55 samples used in this study are available at the Gene Expression Omnibus (GEO) accessible through GEO series accession number: GSE44675. The subset of 23 samples used for the direct comparion of the two enzymes are available in GEO with the series accession number: GSE44641.

### mRNA Sequencing

Total RNA concentration was determined by Qubit high sensitivity spectrofluorometric assay. The polyA RNAs in the total RNA samples (350 to 900 ng) were selected, sheared, and reverse transcribed using the TruSeq RNA library preparation kit. Each sample was fitted with one of 12 adapters containing a different 6 base molecular barcode to allow pooling of multiple samples during sequencing. After 12 cycles of PCR amplification, completed libraries were sequenced on an Illumina HiSeq2000, generating 10 to 20 million of high quality 50 base-long reads per sample.

mRNA-Seq reads were aligned to the mm9 version of the mouse genome reference using the TopHat/Cufflinks pipeline. First, sequences were aligned to the genome with TopHat [79], which efficiently aligned reads spanning known or novel splice junctions. Each sample was then independently processed with Cufflinks [23] in order to generate an initial transcriptome. Finally, the Cuffmerge tool was used to merge the known and any novel isoforms into a single BAM file, and simultaneously extended partial transcripts [80].

### Microarray data normalization and analyses

Methods for microarray analyses were described previously [14]. Data analyses were performed using Partek Genomics Suite Version 6.4 (Partek, St Louis, MO, USA). The Affymetrix Mouse Gene 1.0 ST chip data for the 9V/null mouse with 3 tissue types of 3 different treatments and corresponding WT untreated controls were loaded into Partek Genomics Suite 6.4 (Partek, Inc., St Louis, MO). Normalization was performed using the RMA (robust multiarray average) algorithm [78]. Sample relationships were examined using Principal Components Analyses (PCA) to reveal outliers. The outliers were removed from subsequent analysis. Post normalization and PCA a Mixed Model ANOVA was applied to all the qualifying samples in Partek Genomic suite to identify DEGs between the groups. The contrast comparisons: imig vs. WT control, vela vs. WT control and saline vs. WT control. The genes at ≥1.5 fold change (FC) and a false discovery rate (FDR) of 0.05 were considered as differentially expressed between groups. FDR was used to further guard against false positives because of multiple testing [81].

### Exploratory analyses in mRNA-Seq data sets

Post Binary Alignment/Map (BAM) aligned files of mRNA-Seq data were uploaded in Avadis NGS software. Data analysis was performed using Avadis® NGS software, Version 1.3.0. Reads were filtered to remove a) duplicate reads, b) non-primary-matched reads and c) reads with alignment scores <95. Quantification was performed on the filtered reads against the RefSeq annotation. The initial number of reads was 632783594, which dropped to 292320998 post filtering amounting to 46.19% of the original read counts. PCA and multivariate correlations were performed to access reproducibility and variability among biological replicates. The outliers identified were subsequently removed.

### DEGs analyses with mRNA-Seq data sets

DESeq and edgeR were used to evaluate the DEGs from the mRNA-Seq data. DESeq via R script was performed on the filtered reads by Avadis NGS software using three functions (estimate size factors, estimate dispersions and negative binomial test).

For DESeq normalization, the sequencing depth is estimated by the read count of the gene with the median read count ratio across all genes. The normalized counts are computed:

- For each sample Sj, the normalization factor Nj is calculated as the median of the values r^'^_ij_ where r^'^_ij_ = r_ij_/m_i_. r_ij_ is the read count of gene g_i_ in sample Sj. For each gene g_i_, the geometric mean of the read counts of all the samples for that gene is calculated. Let it be m_i_. While computing this median, the genes with m_i_ = 0 are ignored.
- Finally the normalized counts n_ij_ for gene g_i_ in sample Sj are computed as r_ij_/Nj.

The method is based on the negative binomial distribution, which allows for less restrictive variance parameter assumptions than does the Poisson distribution [32]. Negative binomial (NB) distribution is commonly used to model count data when over dispersion is present [82].

The threshold for detection of the DEGs was set at ±1.5 FC and a FDR of 0.05. Imig, vela, and saline-treated samples from 9V/null spleen, liver and lung were compared with their age and strain matched untreated WT tissue. edgeR (Empirical analysis of digital gene expression data in R) is available from Bioconductor at the URL: http://www.bioconductor.org/packages/2.11/bioc/html/edgeR.html. edgeR is based on a NB underlying distribution to account for variability of replicates on gene-wise dispersions. The Trimmed Mean of M value normalization (TMM) method is incorporated in the package, where different library sizes across samples are adjusted with scaling factors prior to DE analyses to avoid biased detection. DE analysis in edgeR includes two factors in the generalized linear model (glmFit), the treatment specific effect (main factor) and a nuisance factor, to address the variation in the biological replicates which may affect the measured changes of gene expression, and therefore needs to be controlled in the model. Under the null hypothesis, there is no significant difference in change of expression for each tissue between two different biopharmaceuticals for a given gene; glmLRT function is next performed to carry out the likelihood ratio test in edgeR.

### Correlation between mRNA expression levels measured by microarray and mRNA-Seq

Correlations between the signals obtained from microarray and the mRNA-Seq data were evaluated with two-way Venn diagrams on the RefSeq genes common to both platforms and above the detection threshold. The genes in the overlapping region were selected for performing the scatterplot.

Scatter plots were developed from log-transformed intensity and read count values of the selected genes common to the microarray and mRNA-Seq in liver, lung, and spleen samples. Correlation plots on FC values were generated on the DEGs common to all three DE methods in liver, lung and spleen tissues. Correlation between the two platforms and the correlation of the FC values between the three DE methods was evaluated by the Pearson's correlation coefficient and statistical significance with the JMP5 Genomics software.

### Identification of core DEGs

Various statistical algorithms have been developed for identifying DEGs between different groups. Each of these methods use different strategies and thus identify different gene set, which overlap in part. The choice of proper method can have a profound influence on the DEGs identified. We expect that the genes identified by more than one method/algorithm are more likely to be true DEGs. As such our final lists of DEGs were identified in at least two of the algorithms. This approach should result in a more consistent set of DEGs than relying on a single algorithm. The core DEGs are the DEGs which overlap between any two methods.

To identify the core DEGs, a three-way proportional Venn diagram was developed with each circle representing the DEGs determined by one of the DE methods. The overlapping region between any two circles represented the core DEGs. The total number of core DEGs were arrived at by combining the DEGs in all the overlapping regions. These core DEGs were used for functional analyses.

### Functional classification of DEGs

The functional classifications were performed using the Gene Ontology (GO) classification obtained through the DAVID Bioinformatics Resources 6.7 [83], [84] available at http://david.abcc.ncifcrf.gov/home.jsp, ToppCluster, and public information and literature references. For DAVID, the hypergeometric distribution was performed to detect the referenced significant functional categories (*p*<0.05). For ToppCluster a p≤0.05 was chosen as the cutoff value.

The functional analyses were done in two stages as described in Fig. S1. In the first stage the DEGs identified by the three different DE methods under different treatment or no treatment conditions were used directly to identify biological functions. This was performed to evaluate the common and unique features of the functional groups associated with the DEGs identified with each DE methods. For the second stage of functional analyses, the core DEGs for each tissue were determined from the Venn diagrams. The core DEGs was loaded into IPA to identify biological functions, transcriptional regulators, pathways, and global networks. Fisher's exact test with a threshold P≤0.05 was used as a cutoff to identify significant functions and pathways. The functional groups identified from the core DEGs had fewer numbers of false positives. The pathways and networks were constructed from published literature and the IPA database.

### Identification of DEGs between imig and vela treated samples

For direct comparison a total of 23 RNA sample sets were analyzed: 8 lung sets including 4 (imig) and 4 (vela); 8 liver sets: 4 (imig) and 4 (vela); 7 spleen sets: 4 (imig) and 3 (vela), and were interrogated using the microarray and mRNA-Sequencing as described above. To identify the differential expression between the drugs, imig and vela, imig-treated samples were compared with their vela-treated counterparts in the liver, lung and spleen. Mixed Model ANOVA (microarray) and DESeq (mRNA-Seq) were used to identify DEGs of imig/vela, a ±1.5 FC with an FDR of 0.05 was used as selection criteria. GO and network analysis was performed in the DEGs as described above.

## Supporting Information

Figure S1 **Flowchart of microarray and mRNA-Seq data analysis methodology.** The analysis performed simultaneously on two platforms, microarray and mRNA-Seq, to identify DEGs and associated biological functions.
(TIF)

Figure S2 **Principal Component Analysis (PCA)**. a) Eight outliers were identified from total 55 sample sets from the microarray data. b) Four outliers out of 55 sample sets were found in mRNA-Seq data. Green circles indicate the outliers. PCA was applied to assess the variables in the data set. The proportion of variables in each component (X, Y or Z axis) shown under the graph. The addition of three components yielded 82.1% for microarray data and 67.6% for mRNA-Seq data of variation in measure correlations. The first principal component accounts for as much of the variability in the data as possible, the linear combination of X-variables that has maximum variance (among all linear combinations), and each succeeding component accounts for as much of the remaining variability as possible.
(TIF)

Figure S3 **Comparisons of the liver DEGs between microarray and mRNA-Seq.** DEGs were identified by Mixed Model ANOVA (microarray) and DESeq and edgeR (mRNA-Seq). The colors correspond to the analytic methods. (a) saline-treated, (b) imig-treated, (c) vela-treated 9V/null livers. d) The number of DEGs in 9V/null liver identified by DE methods in imig-, vela- and saline-treatment. The genes with increased expression level are shown in dark grey and the genes with decreased expression level are in light grey.
(TIF)

Figure S4 **Comparison of the lung DEGs between microarray and mRNA-Seq by Venn diagrams.** DEGs were identified by Mixed Model ANOVA (microarray) and DESeq and edgeR (mRNA-Seq). The colors correspond to each method. (a) saline-treated, (b) imig-treated and (c) vela-treated 9V/null lung. d) The number of DEGs in 9V/null lung identified by DE methods in imig-, vela- and saline-treatment. The genes with increased expression level are shown in dark grey and the genes with decreased expression level in light grey.
(TIF)

Table S1 (XLSX)

Table S2 (XLSX)

Table S3 (XLSX)

Table S4 (XLSX)

Table S5 (XLSX)

Table S6 (XLSX)

Table S7 (XLSX)

Table S8 (XLSX)

Table S9 (XLSX)

Table S10 (XLSX)

Table S11 (XLSX)

Table S12 (XLSX)

Table S13 (XLSX)

Table S14 (XLSX)
