# Supplementary figures and images for: Gaucher Disease: Transcriptome Analyses Using Microarray or mRNA Sequencing in a Gba1 Mutant Mouse Model Treated with Velaglucerase alfa or Imiglucerase
Source: PLoS One. 2013 Oct 4;8(10):e74912. doi: 10.1371/journal.pone.0074912 (PMC3790783; doi:10.1371/journal.pone.0074912)

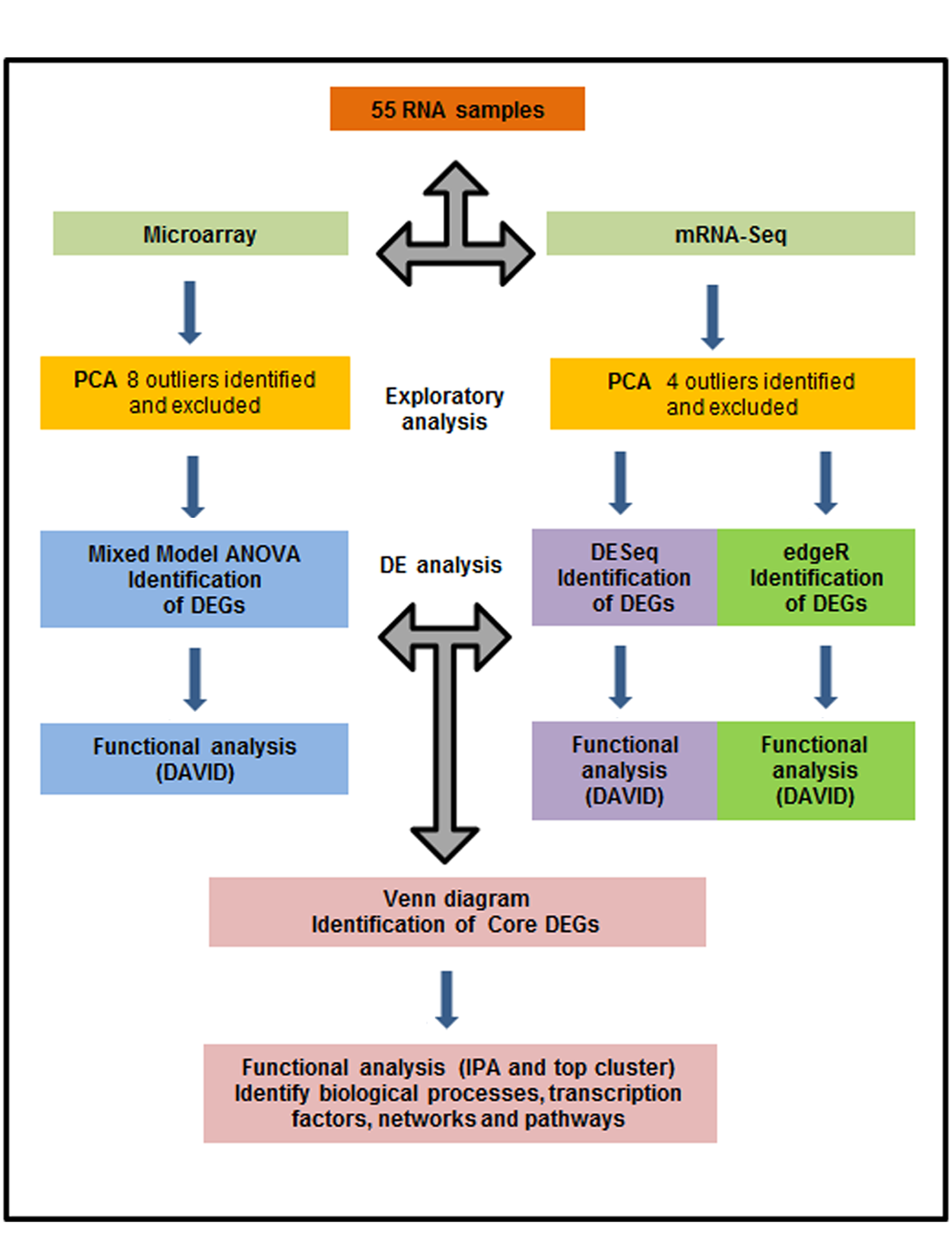

Supplement: Figure S1 — Flowchart of microarray and mRNA-Seq data analysis methodology. The analysis performed simultaneously on two platforms, microarray and mRNA-Seq, to identify DEGs and associated biological functions. (TIF) [file pone.0074912.s001.tif]

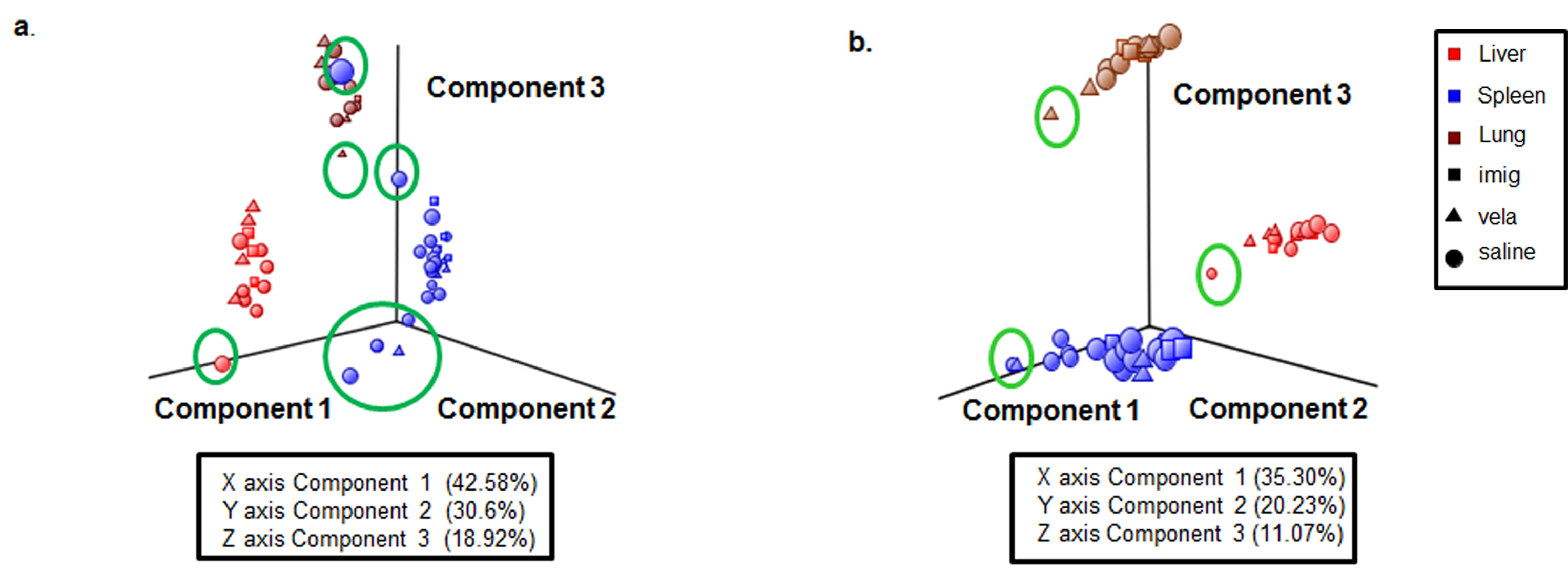

Supplement: Figure S2 — Principal Component Analysis (PCA). a) Eight outliers were identified from total 55 sample sets from the microarray data. b) Four outliers out of 55 sample sets were found in mRNA-Seq data. Green circles indicate the outliers. PCA was applied to assess the variables in the data set. The proportion of variables in each component (X, Y or Z axis) shown under the graph. The addition of three components yielded 82.1% for microarray data and 67.6% for mRNA-Seq data of variation in measure correlations. The first principal component accounts for as much of the variability in the data as possible, the linear combination of X-variables that has maximum variance (among all linear combinations), and each succeeding component accounts for as much of the remaining variability as possible. (TIF) [file pone.0074912.s002.tif]

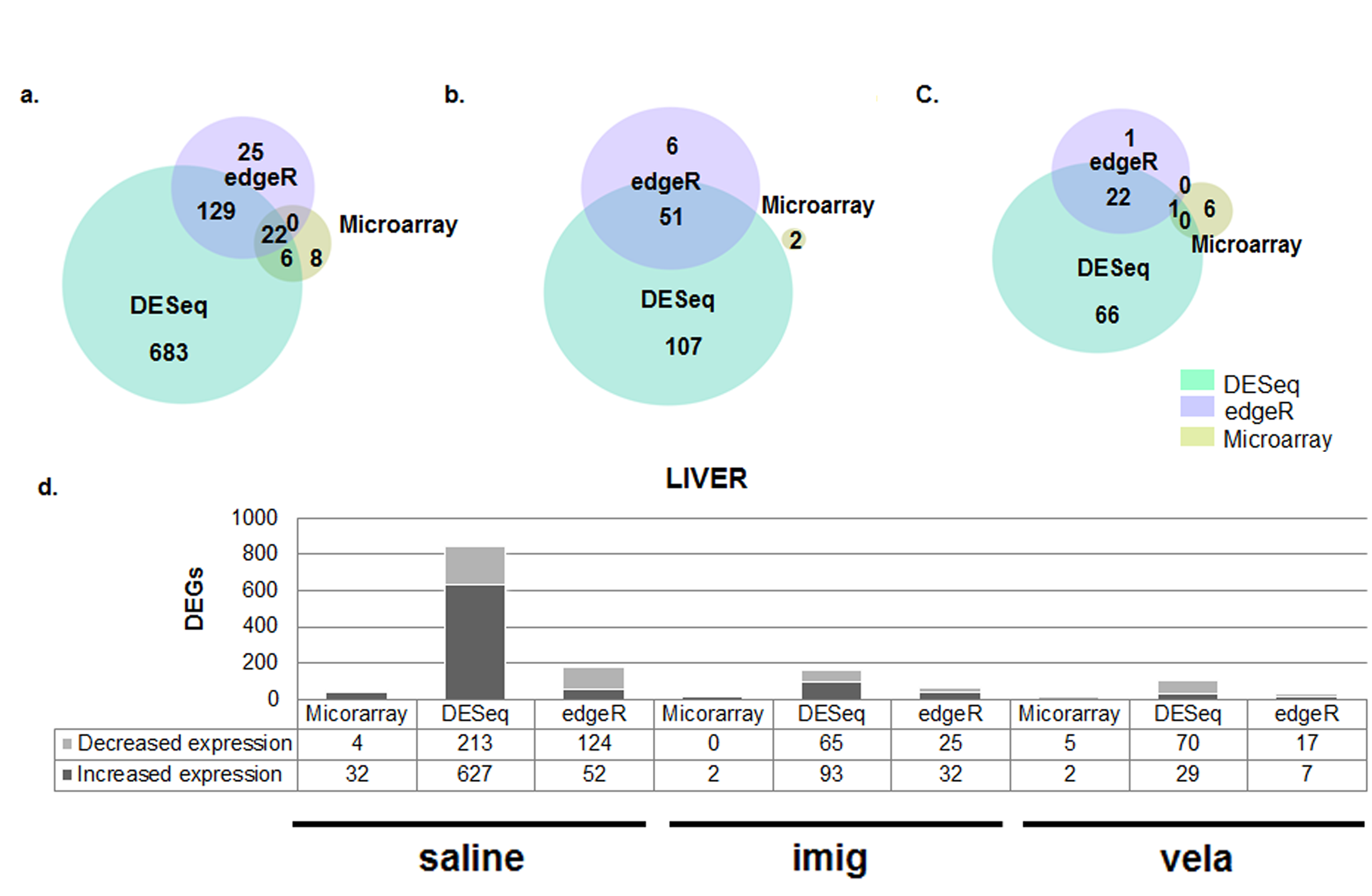

Supplement: Figure S3 — Comparisons of the liver DEGs between microarray and mRNA-Seq. DEGs were identified by Mixed Model ANOVA (microarray) and DESeq and edgeR (mRNA-Seq). The colors correspond to the analytic methods. (a) saline-treated, (b) imig-treated, (c) vela-treated 9V/null livers. d) The number of DEGs in 9V/null liver identified by DE methods in imig-, vela- and saline-treatment. The genes with increased expression level are shown in dark grey and the genes with decreased expression level are in light grey. (TIF) [file pone.0074912.s003.tif]

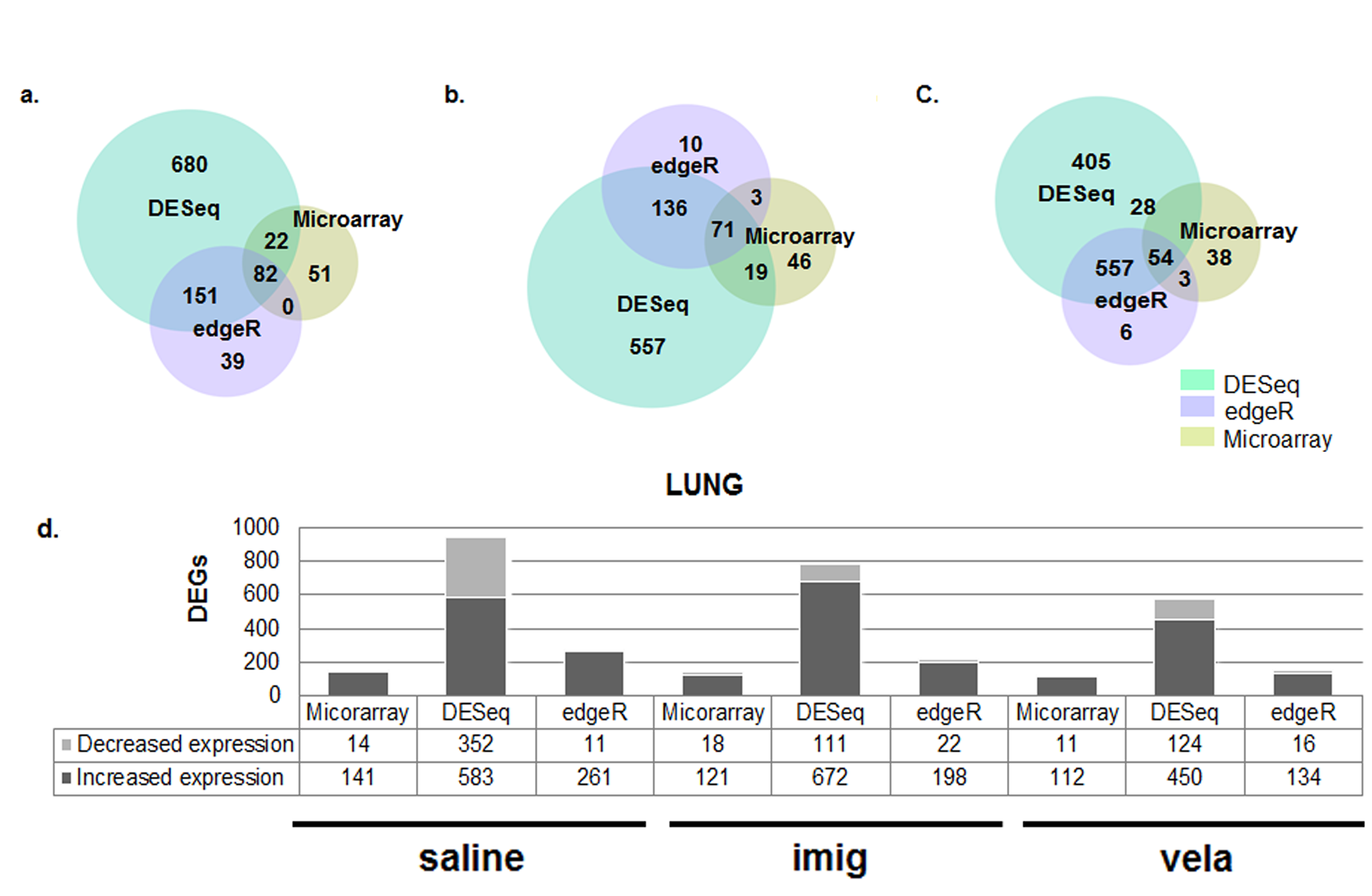

Supplement: Figure S4 — Comparison of the lung DEGs between microarray and mRNA-Seq by Venn diagrams. DEGs were identified by Mixed Model ANOVA (microarray) and DESeq and edgeR (mRNA-Seq). The colors correspond to each method. (a) saline-treated, (b) imig-treated and (c) vela-treated 9V/null lung. d) The number of DEGs in 9V/null lung identified by DE methods in imig-, vela- and saline-treatment. The genes with increased expression level are shown in dark grey and the genes with decreased expression level in light grey. (TIF) [file pone.0074912.s004.tif]
